# Influence of Rolfing Structural Integration on Lower Limb Mobility, Respiratory Thorax Mobility, and Trunk Symmetry: A Retrospective Cohort Study

**DOI:** 10.3390/jcm14176123

**Published:** 2025-08-29

**Authors:** Robert Schleip, Helen James, Katja Bartsch, Eric Jacobsen, David Lesondak, Marilyn E. Miller, Andreas Brandl

**Affiliations:** 1 Conservative and Rehabilitative Orthopedics, TUM School of Medicine and Health, Technical University of Munich, 80333 Munich, Germany; katja.bartsch@tum.de (K.B.);; 2 Department for Medical Professions, Diploma Hochschule, 37242 Bad Sooden-Allendorf, Germany; 3 Department of Physical Therapy, California State University, Fresno, CA 93740, USA; 4 Department of Global Health and Social Medicine, Harvard Medical School, Boston, MA 02115, USA; eric_jacobson@hms.harvard.edu; 5 Department of Family and Community Medicine, University of Pittsburgh Medical Center (UPMC), Pittsburgh, PA 15219, USA; lesondakda@upmc.edu; 6 Department of Physical Therapy, University of St. Augustine for Health Sciences at San Diego, San Diego, CA 92069, USA

**Keywords:** fascia, Rolfing structural integration, manual mobilization

## Abstract

**Background**: Previous research highlights the potential of Rolfing structural integration (SI)—a force-based mobilization of fascia—in modifying postural alignment and joint mobility. This retrospective cohort study builds upon prior work to explore the influence of SI on lower limb mobility, trunk symmetry, and respiratory thoracic expansion. **Methods**: We conducted a retrospective secondary analysis of data drawn from the archive of clinical records as in our previous publication. A total of 563 subjects (aged 18–60 years, BMI 19–29) who completed 10 SI sessions were included. Outcomes evaluated included passive hip flexion (right/left), passive knee flexion mobility (right/left), trunk length symmetry, and chest diameter at normal breath as well as in full inspiration. Wilcoxon signed-rank tests were used for statistical analysis. **Results**: All parameters showed statistically significant improvements post-intervention (*p* < 0.001), including increased thoracic expansion, enhanced trunk symmetry, and improved mobility in hip joint flexion and knee flexion. **Conclusions**: Ten sessions of SI were associated with statistically significant improvements in lower limb mobility, trunk symmetry, and respiratory thoracic mobility. These findings support the role of SI in addressing postural and mobility-related dysfunctions through fascia-oriented mobilization.

## 1. Introduction

Force-based mobilizations of fascia have gained increasing attention for their capacity to alter musculoskeletal and postural dynamics [1]. Rolfing structural integration (SI)—with “Rolfing” being a registered service mark of the Dr. Ida Rolf Institute—is a systematic approach to bodywork aimed at restoring functional alignment and movement efficiency by fascia-oriented manual mobilization [2]. Recent studies highlight the capacity of SI to improve joint range of motion (ROM), suggesting that fascial densification and misalignment may contribute to functional restrictions [3,4,5].

In a recent study by Brandl et al. [4], significant improvements in shoulder and hip ROM were observed following SI. Building on that work, the present study explores additional parameters—lower limb mobility, trunk symmetry, and thoracic expansion using the same retrospective cohort. Thoracic expansion was assessed via normal breathing and full inspiration chest diameter. Trunk length symmetry was assessed in terms of potential side differences in the distance between axilla and iliac crest. Hip flexion and knee flexion mobility assessments were adapted from Janda’s manual testing guidelines [6].

This study was motivated both by a growing body of anecdotal clinical observations and a lack of rigorous large-scale studies on the long-term practice of SI in private health care settings. Given the increasing clinical interest in fascia-oriented therapies, this research aims to contribute to the evidence base supporting SI as a potential complementary intervention for improving mobility and postural symmetry. Understanding its effects in real-world settings could have implications for both clinical rehabilitation and preventive musculoskeletal care.

## 2. Materials and Methods

### 2.1. Study Design and Participants

This study utilized a secondary analysis of the patient records originally evaluated in Brandl et al. [4], which was based on a dataset of 727 records from a 23-year private SI practice. While the first publication focused on fully available data collected from treated patients in the clinic, this article also considers parameters that were only partially surveyed. All participants completed the standard 10-session SI protocol. However, not all parameters were assessed at baseline and at the final assessment of each treated individual. Consequently, the numbers of participants studied per parameter were as follows: chest circumference at normal breathing, chest circumference at full inspiration, *n* = 485; trunk length, *n* = 295; passive hip flexion, *n* = 193; passive knee flexion, *n* = 165.

### 2.2. Inclusion Criteria

Inclusion criteria for this secondary analysis were as follows: (a) completion of 10 SI sessions; (b) male or female subjects aged between 18 and 60 years; (c) a body mass index (BMI) between 19 and 29; and (d) availability of complete pre- and post-intervention data for passive hip flexion and knee flexion mobility on both sides, trunk length (right and left), as well as chest circumference at normal breathing and at full inspiration.

While the included participants ranged in age from 18 to 60 years, no age-based subgroup analyses were conducted within the data archive used for this study. This heterogeneity may limit the ability to detect age-specific effects and should be considered when interpreting the results.

Information regarding participant comorbidities was not systematically recorded in the original data archive. Consequently, no formal exclusion criteria were applied based on existing medical conditions. While this reflects the real-world clinical practice setting in which the data were collected, it also introduces a potential source of heterogeneity and uncontrolled confounding, which is acknowledged in the limitations of this study.

### 2.3. Structural Integration Intervention

The intervention consisted of the standard 10-session Rolfing SI protocol as taught by the Dr. Ida Rolf Institute (www.rolf.org, accessed on 25 May 2025). Each session focused on specific anatomical and functional goals aimed at enhancing structural balance and movement economy [7].

### 2.4. Materials and Measures

Measurements were extracted from clinical records that were routinely documented during the initial and final assessments, conducted before and after completion of the 10-session SI series. All assessments were performed by the same licensed physical therapist who also administered the SI sessions. This practitioner had over 20 years of clinical experience and regularly taught these assessment procedures in a professional educational context. While this ensured consistency, it also introduces the potential for measurement bias, which is acknowledged in the limitations.

All examinations were conducted by a licensed physical therapist and certified Structural Integration (SI) practitioner with more than 20 years of clinical experience. The assessments followed standardized manual procedures and adhered to established guidelines and anthropometric protocols where applicable.

#### 2.4.1. Passive Hip Flexion Mobility (PHF)

**Figure 1:**
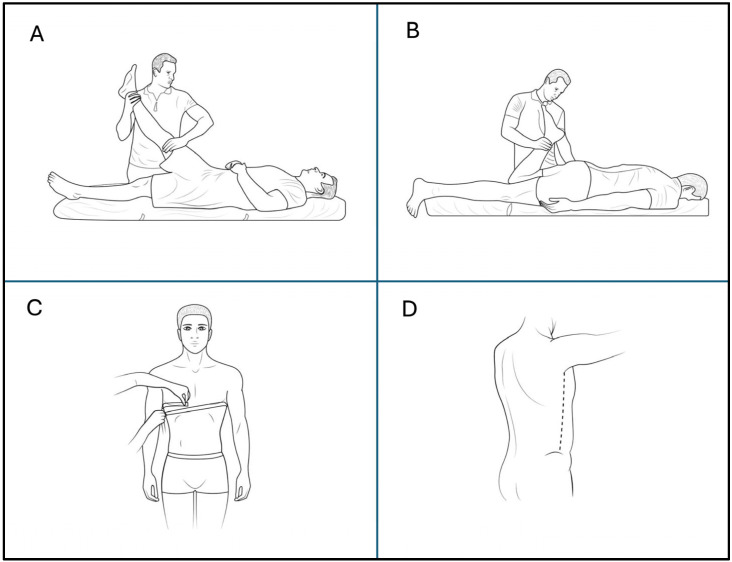
Overview of measurement procedures. (**A**) Passive hip flexion (PHF) assessed in supine position using a goniometer, with the end range defined by the onset of pelvic compensation. (**B**) Passive knee flexion (PKF) measured in prone position, with end range defined by firm resistance or pelvic movement. (**C**) Chest circumference measurement at the level of the 4th intercostal space during normal breathing and full inspiration, performed with a flexible measuring tape positioned horizontally around the thorax. (**D**) Trunk length measurement from axilla to iliac crest on each side, recorded in standing position for assessing trunk length symmetry.

Passive hip flexion was assessed with the patient in a supine position. One leg was raised passively by the examiner, maintaining knee extension throughout the maneuver. The end range of motion was determined by the onset of a visible compensatory movement in the pelvis, such as posterior pelvic tilt or rotation, which indicated the limit of passive hip flexion. A 12-inch, 360° goniometer labeled in 1° increments was used to record the angle of hip flexion. Goniometer alignment followed the procedure described by Elson and Aspinall [8], with the axis positioned at the greater trochanter and the arms aligned with the lateral femoral condyle and the mid-axillary line (Figure 1A).

#### 2.4.2. Passive Knee Flexion Mobility (PKF)

Passive knee flexion was measured with the patient in a prone position. The examiner lifted the lower leg passively by flexing the knee while stabilizing the pelvis. The end range was identified either by a firm resistance to motion or by the appearance of compensatory pelvic movement (e.g., anterior pelvic tilt). Knee joint angle was measured using a goniometer, with the axis placed at the lateral epicondyle of the femur and the arms aligned with the greater trochanter and the lateral malleolus. This procedure followed the recommendations by Janda [6] (Figure 1B).

#### 2.4.3. Chest Circumference at Normal Breathing (CC-NB)

Chest circumference at normal breathing was measured with the subject standing upright and breathing calmly. The measurement was taken at the end of a relaxed expiration. A flexible, non-elastic tape was wrapped horizontally around the thorax at the level of the fourth intercostal space—approximately the nipple line in men and just below the breasts in women—crossing over the sternum anteriorly and the inferior angle of the scapula posteriorly. This procedure followed the recommendations for respiratory anthropometry described by the Centers for Disease Control and Prevention [9] (Figure 1C).

#### 2.4.4. Chest Circumference at Full Inspiration (CC-FI)

Maximal chest expansion was assessed using the same tape placement as described above. Subjects were instructed to inhale maximally, and the measurement was taken at the peak of full inspiration. The measurement procedure adhered to CDC anthropometric protocols (Centers for Disease Control and Prevention, 1988) and ensured consistency with the normal breathing assessment.

#### 2.4.5. Trunk Length Symmetry (TLS)

Trunk length was assessed bilaterally as the vertical distance between the axilla and the ipsilateral iliac crest, with the patient standing in an upright, relaxed posture. Measurements were taken using a flexible anthropometric tape, in accordance with established protocols described in the NASA Anthropometric Source Book [10]. Right and left side measurements were recorded separately, and trunk length symmetry was calculated as the absolute difference between the two (Figure 1D).

### 2.5. Statistical Analysis

After inspection of the Q-Q plots and Shapiro-Wilk normality test (all *p* < 0.001), the parameters did not meet the criteria for parametric testing. Therefore, non-parametric Wilcoxon signed-rank tests with rank–biserial correlations as effect sizes, medians, and interquartile differences were performed to compare the measures before and after the intervention. A gender-specific subgroup analysis was conducted, and the differences at the measurement times were analyzed using the Mann–Whitney U test.

*p* values were corrected by the False Discovery Rate Correction [11]. The effect sizes were interpreted according to Cohen as small (r < 0.3), medium (r ≥ 0.3, <0.5), and large (r ≥ 0.5). The significance level was set at *p* = 0.05.

All statistics were carried out with the software R, version 3.4.1 (R Foundation for Statis-tical Computing, Vienna, Austria).

## 3. Results

Table 1 displays the baseline characteristics and anthropometric data. A total of 497 patients treated between 2 July 1982 and 18 November 2005 satisfied the requirements for eligibility and were examined.

**Figure 2:**
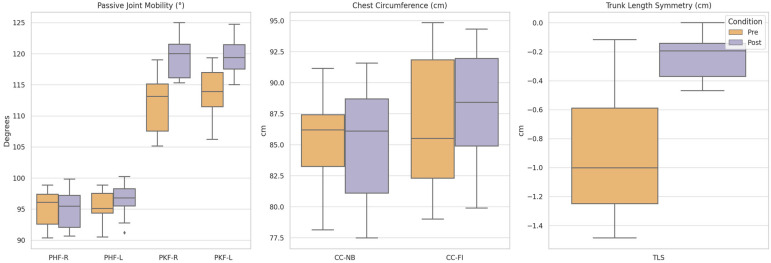
Pre- and post-treatment comparisons across all measured parameters. Box plots display the distribution of measurement values before and after Rolfing Structural Integration (SI) treatment for each outcome variable. The data within the boxes refers to the 2nd to 3rd quartile. The horizontal lines mark the median. The whiskers show the 1.5-fold interquartile range from the 2nd to 3rd quartiles. Other points mark outliers. PHF = Passive Hip Flexion; PKF = Passive Knee Flexion; CC-NB = Chest Circumference at Normal Breathing; CC-FI = Chest Circumference at Full Inspiration; TL = Trunk Length. Significant improvements were observed in all parameters by Wilcoxon signed-rank test with False Discovery Rate correction. The plots illustrate consistent pre- to post-increases in mobility and reductions in trunk asymmetry.

Significant improvements were observed across all measured parameters following the Rolfing SI intervention (Table 2, Figure 2). Passive joint mobility increased bilaterally in both the hip and knee joints, thoracic respiratory mobility improved in both normal and deep inspiration, and trunk length asymmetry was reduced (Table 3).

## 4. Discussion

This secondary analysis of the study data from Brandl et al. [4] aimed to provide comprehensive information on the impact of SI on patients’ health, with emphasis on musculoskeletal and respiratory functioning. This retrospective study, along with the work of James et al. [3], was the first to examine SI in 723 subjects in the specific health care setting of a private practice over a representative 23-year period.

This novel analysis focuses on data that were incomplete in the original dataset. It is possible that in daily manual treatment practice, results that are not frequently collected are considered less important than others (i.e., those published by Brandl et al. [4]). However, the parameters now studied were still available in large numbers (*n* from 165 to 492), and not publishing all available data from a dataset may pose some risk of publication bias.

The presented statistical analysis of these novel data suggests that SI has measurable effects on lower limb and thoracic respiratory mobility, as well as on trunk length symmetry. Enhancements in thoracic expansion mobility may facilitate improved respiratory function, while gains in lower limb mobility and in trunk length symmetry may reflect improved biomechanical alignment. These changes may be due to improved myofascial gliding, altered neuromuscular tone, and psychoneurobiological influences, as hypothesized in prior publications [5,12,13,14].

### 4.1. Functional and Myofascial Mechanisms Underlying the New Findings

The results of this study extend the findings of our previous analysis [4], suggesting that Rolfing SI may influence additional aspects of musculoskeletal function beyond cervical and shoulder mobility. Specifically, we observed improvements in lower limb mobility (e.g., passive hip and knee flexion), thoracic respiratory mobility, and trunk length symmetry. These findings align with the hypothesis that SI exerts system-wide effects through mechanical, neurological, and potentially psychobiological mechanisms.

The observed functional improvements are consistent with outcomes reported in other fascia-based manual therapies, such as Myofascial Release and Osteopathic Manipulative Treatment [5,15,16]. These approaches have similarly demonstrated effects on joint range of motion, postural alignment, and respiratory function, reinforcing the hypothesis that manual fascial interventions can induce system-wide adaptations.

Previous research has shown that force transmission occurs across myofascial chains, particularly along the posterior kinetic chain [17,18]. As noted in our earlier study [4], both cadaveric and in vivo investigations demonstrate that tension can propagate from the plantar fascia through the gastrocnemius, hamstrings, and thoracolumbar fascia to the paraspinal muscles. Improvements in hip and knee mobility may therefore reflect changes in these fascial continuities, possibly mediated through reduced tissue stiffness or restored sliding capacity between fascial planes, neural pathways, and adjacent structures [1].

Of particular interest is the observed increase in thoracic expansion during maximal inspiration. This may indicate both local improvements in the pliability of intercostal and costovertebral structures, and broader changes in postural tone and rib mobility. Manual therapies such as Myofascial Release or Osteopathic Manipulative Treatment have been shown to affect thoracic and pelvic shape parameters [15,16,19], often through rapid neuromuscular effects. Brandl et al. [19] examined the influence of myofascial release therapy on the pelvic obliquity and found a reduction of 5.2 mm after one single treatment. It is plausible that SI exerts similar influences, but in this study potentially with longer-lasting outcomes due to its integrated use of hands-on techniques and guided movement retraining over 10 treatment sessions.

The reduction in trunk length asymmetry may reflect improvements in spinal alignment, lateral fascial balance, or neuromotor control. These observations support the notion that SI affects not only isolated joint mobility, but also global body organization and postural symmetry. Mechanistically, such effects may be mediated by input from mechanoreceptors embedded in deep fascial tissues—such as the fascia profunda, tendons, joint capsules, and intramuscular connective tissue—which in turn modulate muscle tone, tissue hydration, and sensory–motor coordination [20,21].

While these mechanisms remain speculative, they are consistent with emerging biopsychosocial models of manual therapy that highlight its multisystem effects [14,22,23]. As discussed previously [4], it is difficult to isolate the impact of soft tissue manipulation from that of movement education, attentional shifts, or the therapeutic alliance. Nonetheless, the present results add to the growing body of evidence suggesting that SI may elicit integrated structural and functional adaptations across multiple systems.

### 4.2. Interpreting Functional Gains and Clinical Impact

These results are supported not only by statistically significant changes but also by meaningful effect sizes, suggesting clinical relevance. Passive hip and knee flexion demonstrated large effect sizes (r > 0.6 and r > 0.8, respectively), consistent with substantial functional gains in lower limb mobility. Such magnitudes exceed what is often considered a minimal clinically important difference (MCID) in musculoskeletal interventions [24,25], underscoring the potential relevance of these findings in a rehabilitative context. However, while the MCID of 3.8° for passive knee flexion [26] was exceeded in our study, the MCID for passive hip flexion remained below the value of 3.29° [27], and the clinical utility of the statistically large effect needs to be further investigated in subsequent controlled studies.

Thoracic expansion at full inspiration also reached a medium-to-large effect size (r ≈ 0.55), which may reflect improved respiratory efficiency or thoracic compliance. While normative MCID values for chest expansion are less clearly established, changes of 3–5 cm are typically considered functionally meaningful in respiratory therapy [28]. The moderate effect sizes observed in trunk length symmetry (r ≈ 0.45–0.48) suggest that SI may reduce structural asymmetry or functional lateral imbalance, both of which are relevant to spinal health and movement coordination.

Although chest circumference during normal breathing showed only a small effect (r ≈ 0.1), even modest improvements in baseline respiratory parameters may be clinically relevant, particularly in populations with restrictive or age-related mobility deficits.

A sex-specific subgroup analysis revealed differences for most parameters, which is consistent with current knowledge that range of motion is significantly sex-dependent [29]. However, the changes due to SI in this study were significant in both groups, women and men, so we assume that the effects of SI in this study were cross-sex.

Taken together, these results suggest that SI may provide multifactorial benefits—not only in segmental joint mobility but also in postural and respiratory function—warranting further investigation in controlled prospective studies with standardized clinical outcomes.

### 4.3. Limitations

While the longitudinal data acquisition over a 23-year period enhances the external validity of our findings, the retrospective and uncontrolled nature of the study imposes important methodological limitations.

As in the initial study using this data set, caution is warranted when interpreting the results due to the absence of a control group. Spontaneous improvements, placebo effects, or concurrent lifestyle changes may have contributed to the observed outcomes. Given this context, no definitive conclusions can be drawn regarding specific causal relationships between the reported parameters. Although the evaluator remained the same individual—who was also involved in teaching the respective assessment procedures in a professional educational context—it is important to acknowledge that the retrospective nature of the data archive, spanning more than two decades, limits control over confounding variables, potential inconsistencies in measurement, and possible biases in record-keeping.

The use of this large data archive—based on self-selected participants and characterized by uncontrolled demographic and ethnic diversity—limits the generalizability of the results. As no official data are available regarding the typical gender distribution, age, occupation, or baseline symptoms of clients in international SI practice, comparisons with other clinical or research contexts should be made with caution.

As with the prior analysis [4], randomization was not feasible due to the observational design, and the absence of a control group prevents causal inference. A potential selection bias may also be present, as individuals seeking SI may differ systematically from the general population in terms of health awareness, body perception, or treatment preference. In addition, follow-up data were not available, and concurrent therapies during the SI treatment period could not be controlled for.

Pain and disability outcomes were not included in this analysis, consistent with the previous study, which also lacked such data in the original medical records [4]. Therefore, we were unable to assess patient-centered changes such as improvements in quality of life, function, or pain-related disability—outcomes recommended for future studies (e.g., Oswestry Disability Index, Tinetti Performance-Oriented Mobility Assessment, Berg Balance Scale, or Pain Disability Index).

A further limitation is the lack of standardized documentation of participant comorbidities, which may have influenced the outcomes. Future prospective studies should include systematic health status screening to allow for more precise subgroup analyses.

The study did not include a long-term follow-up, leaving the durability of the observed effects unknown. Future research should incorporate longitudinal assessments to address this important aspect.

The wide age range among participants introduces potential variability in outcomes, particularly given age-related differences in mobility and connective tissue properties. Future studies may benefit from stratified analyses or age-matched cohorts.

The archived data records did not include information as to whether participants engaged in other forms of therapy, exercise, or other lifestyle changes during the treatment series. It is therefore possible that such additional interventions could have influenced the outcomes independently from the SI treatment.

The protocol for SI treatments applied over the 23-year span of this study adhered to the basic guidelines described by Findley et al. [7] and was consistently administered by the same SI practitioner. While these guidelines are rooted in the original teachings of Ida Rolf, founder of the Rolfing method of Structural Integration, and do not differ substantially from earlier internal protocols within the method, they do allow for some minor yet meaningful variation in application—both across patients and between different practitioners. Therefore, caution is warranted when extrapolating the findings of this study to other SI settings or practitioners.

The potential explanations we propose for the observed effects—such as the involvement of myofascial force transmission or neuromuscular coordination, as discussed in Section 4.1—are not based on direct measurements taken in this study. They remain purely hypothetical and should not be interpreted as established facts.

Despite these limitations, the inclusion of a large sample over a long period provides valuable descriptive data. While causality cannot be inferred, the results offer hypothesis-generating insights and contribute to a growing but still limited body of evidence on SI. A current review has highlighted the scarcity of research and small sample sizes in studies on Structural Integration and other body-centered interventions [2].

Our findings offer a preliminary basis for future clinical research on Structural Integration, particularly studies that incorporate standardized and clinically meaningful outcome measures. To strengthen the evidence base, prospective controlled trials are warranted to confirm these results and clarify their clinical significance.

## 5. Conclusions

Rolfing SI was associated with measurable improvements in lower limb and thoracic respiratory mobility, as well as in trunk length symmetry. However, given the retrospective nature of the study, the absence of a control group, and the lack of systematic data on potential confounding variables such as comorbidities, caution is warranted in interpreting these findings as causal.

Nevertheless, the observed changes suggest that SI may promote structural and functional improvements through multifactorial mechanisms. These results provide a valuable basis and orientation for future prospective studies aiming to explore the clinical relevance, underlying mechanisms, and long-term effects of SI using controlled designs and standardized outcome measures.

## Figures and Tables

**Table 1:** Baseline characteristics.

Baseline Characteristics	Participants (*n* = 497) Mean ± SD
Sex (men/woman)	176/321
Age (years)	39.0 ± 11.1
Height (m)	1.69 ± 0.1
Weight (kg)	71.4 ± 16.2
BMI (kg/m^2^)	25.0 ± 4.8

SD, standard deviation; *n*, number; BMI, body mass index.

**Table 2:** Significance of pre- versus post-treatment change scores.

		PHF Right	PHF Left	PKF Right	PKF Left	CC-NB	CC-FI	TLS
**All** **participants**	**Wilcoxon W**	2753.0	3655.0	0.0	57.5	47,680.5	22,966.5	8986.0
	*p* (adjusted) ^1^	**<0.001**	**<0.001**	**<0.001**	**<0.001**	**0.026**	**<0.001**	**0.001**
	**Effect size ^2^**	−0.50	−0.34	−1	−0.98	0.13	−0.50	−0.25
**Female**	**Wilcoxon W**	1108.0	1505.5	0.0	42.0	19,832.0	9703.0	3239.0
	*p* (adjusted) ^1^	**<0.001**	**0.013**	**<0.001**	**<0.001**	0.098	**<0.001**	**0.008**
	**Effect size ^2^**	−0.43	−0.30	−1	−0.97	0.12	−0.49	−0.26
**Men**	**Wilcoxon W**	373.0	476.0	0.0	0.0	6018.0	2902.0	1452
	*p* (adjusted) ^1^	**<0.001**	**0.008**	**<0.001**	**<0.001**	0.111	**<0.001**	**0.049**
	**Effect size ^2^**	−0.59	−0.40	−1	−1	0.15	−0.49	−0.24

PHF, passive hip flexion mobility; PKF, passive knee flexion mobility; CC-NB, circumference normal breath; CC-FI, circumference forced inspiration; TLS, trunk length symmetry. ^1^ False Discovery Rate alpha error correction; ^2^ rank biserial correlation. Significant changes are marked in bold.

**Table 3:** Descriptive statistics.

					Percentiles	
	Time	Sex	N	Median	25th	75th
**PHF right (°)**	T1	all	193	94.00	90.00	99.00
		female	115	91.50 *	88.00	95.0
		men	78	99.00	95.38	104.0
	T2	all	193	95.00	90.50	100.0
		female	115	92.50 *	89.00	96.0
		men	78	100.0	95.25	105.0
**PHF left (°)**	T1	all	190	95.00	90.38	99.00
		female	113	92.00 *	89.00	96.0
		men	77	99.20	96.13	104.0
	T2	all	190	95.50	91.00	100.4
		female	113	93.00 *	89.50	97.0
		men	77	100.5	95.50	105.0
**PKF right (°)**	T1	all	165	115.0	105.0	120.0
		female	105	115.0	105.0	120.0
		men	60	115.0	108.8	120.0
	T2	all	165	120.0	115.0	125.0
		female	105	125.0 *	115.0	130.0
		men	60	120.0	115.0	125.0
**PKF left (°)**	T1	all	165	115.0	105.0	120.0
		female	105	115.0	105.0	120.0
		men	60	110.0	105.0	120.0
	T2	all	165	120.0	115.0	125.0
		female	105	120.0 *	115.0	125.0
		men	60	120.0	113.8	121.3
**CC-NB (cm)**	T1	all	485	83.25	76.50	92.50
		female	312	79.00 *	74.00	84.00
		men	172	93.00	87.25	100.5
	T2	all	485	83.00	76.50	92.00
		female	312	78.13 *	74.50	83.6
		men	172	92.50	87.38	100.6
**CC-FI (cm)**	T1	all	484	85.50	79.00	95.00
		female	312	81.50 *	76.50	86.3
		men	172	96.50	90.75	103.0
	T2	all	484	86.00	79.50	95.10
		female	312	81.50 *	78.00	86.6
		men	172	97.00	91.50	104.0
**TLS (cm)**	T1	all	294	−0.50	−1.50	0.00
		female	183	−0.25	−1.25	0.00
		men	111	−0.50	−1.50	0.00
	T2	all	294	0.00	−0.50	0.00
		female	183	0.00	−0.50	0.00
		men	111	0.00	−1.00	0.00

PHF, passive hip flexion mobility; PKF, passive knee flexion mobility; CC-NB, circumference normal breath; CC-FI, circumference forced inspiration; TLS, trunk length symmetry. * Significant sex differences according to the Mann–Whitney U-test, *p* < 0.05.

## Data Availability

Data can be made available by the author upon request.

## Abbreviations

The following abbreviations are used in this manuscript:

BMI	Body mass index
CC-FI	Chest circumference at full inspiration
CC-NB	Chest circumference at normal breathing
MCID	Minimal clinically important difference
PHF	Passive hip flexion mobility
PKF	Passive knee flexion mobility
ROM	Range of motion
SI	Structural Integration
TLS	Tunk length symmetry

## References

[1] Schleip R., Gabbiani G., Wilke J., Naylor I., Hinz B., Zorn A., Jäger H., Breul R., Schreiner S., Klingler W. (2019). Fascia Is Able to Actively Contract and May Thereby Influence Musculoskeletal Dynamics: A Histochemical and Mechanographic Investigation. Front. Physiol..

[2] Jacobson E. (2011). Structural integration: Origins and development. J. Altern. Complement. Med..

[3] James H., Castaneda L., Miller M.E., Findley T. (2009). Rolfing structural integration treatment of cervical spine dysfunction. J. Bodyw. Mov. Ther..

[4] Brandl A., Bartsch K., James H., Miller M.E., Schleip R. (2022). Influence of Rolfing Structural Integration on Active Range of Motion: A Retrospective Cohort Study. J. Clin. Med..

[5] Santos T.S., Oliveira K.K.B., Martins L.V., Vidal A.P.C. (2022). Effects of manual therapy on body posture: Systematic review and meta-analysis. Gait Posture.

[6] Janda V. (1983). Muscle Function Testing.

[7] Findley T., DeFilippis J. (2005). Information for Clinical Health Care Practitioners.

[8] Elson R.A., Aspinall G.R. (2008). Measurement of hip range of flexion-extension and straight-leg raising. Clin. Orthop. Relat. Res..

[9] Centers for Disease Control and Prevention (1988). Anthropometry Procedures Manual.

[10] NASA (1978). NASA Anthropometric Source Book: Volume II—A Handbook of Anthropometric Data.

[11] Benjamini Y., Hochberg Y. (1995). Controlling the False Discovery Rate: A Practical and Powerful Approach to Multiple Testing. J. R. Stat. Soc. Ser. B Stat. Methodol..

[12] Elkjær E., Mikkelsen M.B., Michalak J., Mennin D.S., O’Toole M.S. (2022). Expansive and Contractive Postures and Movement: A Systematic Review and Meta-Analysis. Perspect. Psychol. Sci..

[13] Weinberg R.S., Hunt V.V. (1976). The interrelationships between anxiety, motor performance and electromyography. J. Mot. Behav..

[14] Brandl A., Engel R., Egner C., Schleip R., Schubert C. (2024). Relations between Daily Stressful Events, Exertion, Heart Rate Variability, and Thoracolumbar Fascia Deformability: A Case Report. J. Med. Case Rep..

[15] Tozzi P., Bongiorno D., Vitturini C. (2011). Low back pain and kidney mobility: Local osteopathic fascial manipulation decreases pain perception and improves renal mobility. J. Bodyw. Mov. Ther..

[16] Cathcart E., McSweeney T., Johnston R., Young H., Edwards D.J. (2019). Immediate biomechanical, systemic, and interoceptive effects of myofascial release on the thoracic spine: A randomised controlled trial. J. Bodyw. Mov. Ther..

[17] Krause F., Wilke J., Vogt L., Banzer W. (2016). Intermuscular force transmission along myofascial chains: A systematic review. J. Anat..

[18] Wilke J., Krause F., Vogt L., Banzer W. (2016). What Is Evidence-Based About Myofascial Chains: A Systematic Review. Arch. Phys. Med. Rehabil..

[19] Brandl A., Egner C., Schleip R. (2021). Immediate Effects of Myofascial Release on the Thoracolumbar Fascia and Osteopathic Treatment for Acute Low Back Pain on Spine Shape Parameters: A Randomized, Placebo-Controlled Trial. Life.

[20] Stecco C., Macchi V., Porzionato A., Duparc F., De Caro R. (2011). The fascia: The forgotten structure. Ital. J. Anat. Embryol..

[21] Schleip R., Müller D.G. (2013). Training principles for fascial connective tissues. J. Bodyw. Mov. Ther..

[22] Langevin H.M. (2006). Connective tissue: A body-wide signaling network?. Med. Hypotheses.

[23] Bianchi M., Rossettini G., Cerritelli F., Esteves J.E. (2025). Insights into how manual therapists incorporate the biopsychosocial-enactive model in care of individuals with CLBP. Chiropr. Man. Therap..

[24] Jaeschke R., Singer J., Guyatt G.H. (1989). Measurement of health status: Ascertaining the minimal clinically important difference. Control Clin. Trials.

[25] Copay A.G., Subach B.R., Glassman S.D., Polly D.W., Schuler T.C. (2007). Understanding the minimum clinically important difference. Spine J..

[26] Pantouveris M., Kotsifaki R., Whiteley R. (2024). Inclinometers and Apps Are Better than Goniometers, Measuring Knee Extension Range of Motion in Anterior Cruciate Ligament Patients: Reliability and Minimal Detectable Change for the Three Devices. J. Knee Surg..

[27] Adegoke B.O., Fapojuwo O.A. (2010). Range of Active Hip Motion in Low Back Pain Patients and Apparently Healthy Controls. J. Allied Health Sci. Pract..

[28] Bockenhauer S.E., Chen H., Julliard K.N., Weedon J. (2007). Measuring thoracic excursion: Reliability of the cloth tape measure technique. J. Am. Osteopath. Assoc..

[29] Hwang J., Jung M.-C. (2015). Age and Sex Differences in Ranges of Motion and Motion Patterns. Int. J. Occup. Saf. Ergon..

